# Supplementary material for: Functional and Molecular Characterisation of Heart Failure Progression in Mice and the Role of Myosin Regulatory Light Chains in the Recovery of Cardiac Muscle Function
Source: Int J Mol Sci. 2021 Dec 22;23(1):88. doi: 10.3390/ijms23010088 (PMC8745055; doi:10.3390/ijms23010088)
Supplement: Supplementary file 1 [file ijms-23-00088-s001.zip › ijms-1465911-supplementary.pdf]

Supplementary Materials

Open chest surgery and differences in age do not affect cardiac muscle contractile force

To monitor the progression of heart failure, papillary contraction force at six time points (2D-, 4D-, 7D-, 10D-, 14D- and 28D post-MI surgery) were measured using 4-5 weeks old mice. Two kinds of controls were studied to see the effects of open chest surgery and ageing in cardiac function. The control group were mice that did not undergo any surgery while the sham group had open chest surgery but without LAD ligation. The average contractile force of permeabilised papillary muscle in the control group was 8.15 ± 2.06 kN/m^2 (n=46), and the average contraction force for the sham group was 7.11 ± 0.83 kN/m^2 (n=87). There is no significant difference between these two groups. i.e., the force of contraction between the control and sham group at all time points was identical and no change in force was observed during the 28 days of the experimental periods within the control and sham groups, respectively. Results show that surgery and difference in age (due to 28 days timeline in the experiments) does not affect contraction force in permeabilised papillary muscle. Therefore, the changes reported in the post-MI group is a result of the LAD surgery.

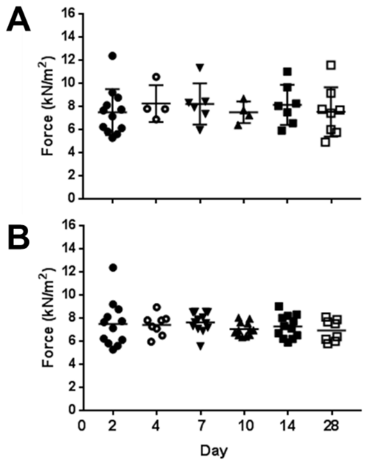

Figure S1. Comparison of the contractile force in the control and sham group. A: Force data of control group. Each point is the average of three measurements from one muscle preparation from the permeabilised papillary. 'n' is 4-13 muscle preparations in each group. B: Force data of sham group. Each point is the average of at least three muscle preparations from permeabilised papillary of one mouse. 'n' is 8-13 preparations in each group. Kruskal-Wallis test was performed on control groups and ordinary one-way ANOVA was performed on sham groups. Both tests no significant differences, respectively.

Table S1. Summary of sarcomeric protein phosphorylation level and maximal isometric force values of post-MI and sham groups during HF progression.

**RLC**	**2D Post-MI**	**2D Sham**	**4D Post-MI**	**4D Sham**	**7D Post-MI**	**7D Sham**	**10D Post-MI**	**10D Sham**	**14D Post-MI**	**14D Sham**	**28D Post-MI**	**28D Sham**
Number of mice	5	3	4	3	3	2	2	3	4	4	3	4
Phospho. Level ± SEM	0.221 ± 0.01	0.369 ± 0.02	0.349 ± 0.02	0.304 ± 0.01	0.42 ± 0.02	0.332 ± 0.001	0.267 ± 0.04	0.353 ± 0.01	0.284 ± 0.01	0.343 ± 0.01	0.287 ± 0.01	0.306 ± 0.01
**MyBP-C**	**2D Post-MI**	**2D Sham**	**4D Post-MI**	**4D Sham**	**7D Post-MI**	**7D Sham**	**10D Post-MI**	**10D Sham**	**14D Post-MI**	**14D Sham**	**28D Post-MI**	**28D Sham**
Number of mice	3	2	3	3	3	3	2	3	3	3	3	3
Phospho. Level ± SEM	1.22 ± 0.07	1.03 ± 0.1	1.08 ± 0.08	1.38 ± 0.02	0.978 ± 0.05	1.05 ± 0.07	0.866 ± 0.009	0.987 ± 0.01	1.41 ± 0.02	1.07 ± 0.1	1.33 ± 0.04	1.09 ± 0.1
**TnI**	**2D Post-MI**	**2D Sham**	**4D Post-MI**	**4D Sham**	**7D Post-MI**	**7D Sham**	**10D Post-MI**	**10D Sham**	**14D Post-MI**	**14D Sham**	**28D Post-MI**	**28D Sham**
Number of mice	3	2	3	3	3	3	2	3	3	2	3	3
Phospho. Level ± SEM	0.989 ± 0.06	1.43 ± 0.05	1.54 ± 0.1	1.55 ± 0.08	1.52 ± 0.1	1.54 ± 0.06	1.49 ± 0.03	1.48 ± 0.03	1.35 ± 0.05	1.63 ± 0.03	1.58 ± 0.06	1.53 ± 0.06
**Isometric Force**	**2D Post-MI**	**2D Sham**	**4D Post-MI**	**4D Sham**	**7D Post-MI**	**7D Sham**	**10D Post-MI**	**10D Sham**	**14D Post-MI**	**14D Sham**	**28D Post-MI**	**28D Sham**
Number of mice	5	4	5	3	5	4	5	3	8	4	6	5
Mean of Max. Isometric Force ± SEM (kN/m^2)	5.06 ± 0.314	7.34 ± 0.255	11.0 ± 0.725	7.18 ± 0.341	7.28 ± 0.704	7.31 ± 0.376	6.38 ± 0.971	7.04 ± 0.277	4.42 ± 0.219	7.25 ± 0.103	4.52 ± 0.529	6.56 ± 0.229

Table S2. Summary of correlations between maximal isometric force and sarcomeric protein phosphorylation and among sarcomeric protein phosphorylation in mice.

**Correlation**	**Sample Size**	**Correlation Coefficient (Spearman r)**	***p* Value**
MyBP-C phosphorylation—Force	*n* = 3 (MyBP-C)<br>*n* = 5–8 (Force)	−0.943 (**)	0.008
Tnl phosphorylation—Force	*n* = 3 (Tnl)<br>*n* = 5–8 (Force)	0.143	0.401
RLC phosphorylation—Force	*n* = 6–17 (RLC)<br>*n* = 5–8 (Force)	0.49	0.178
RLC phosphorylation—MyBP-C	*n* = 6–17 (RLC)<br>*n* = 3 (MyBP-C)	−0.257	0.329
RLC phosphorylation—Tnl	*n* = 6–17 (RLC)<br>*n* = 3 (Tnl)	0.314	0.282

'*n*' refers to the technical replicate at each timepoint, from 2–6 mice. ** $p < 0.01$.

Table S3. Summary of phosphorylation levels and maximal isometric force of 14D post-MI and sham papillary segments before and after RLC exchange.

**RLC Phosphorylation**	**14D Sham (Before Exchange)**	**14D Sham (Exchanged)**	**14D Post-MI (Before Exchange)**	**14D Post-MI (Exchanged)**
Number of mice	2	2	2	2
Phospho. Level ± SEM	0.351 ± 0.00150	0.432 ± 0.00231	0.09 ± 0.0380	0.329 ± 0.00714
**Isometric Force**	**14D Sham (Before Exchange)**	**14D Sham (Exchanged)**	**14D Post-MI (Before Exchange)**	**14D Post-MI (Exchanged)**
Fibre segments measured	6	6	9	9
Max. Isometric Force ± SEM (kN/m2)	6.93 ± 0.457	9.69 ± 0.534	3.95 ± 0.211	6.20 ± 0.218

Relationship between RLC phosphorylation and enzymes in sham groups

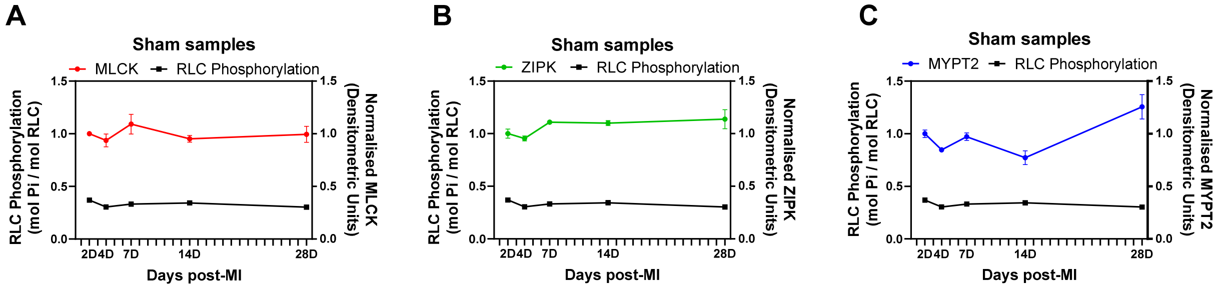

Figure S2: Graphs of RLC phosphorylation and normalised enzyme amounts of sham groups.

Differential expression of GAPDH

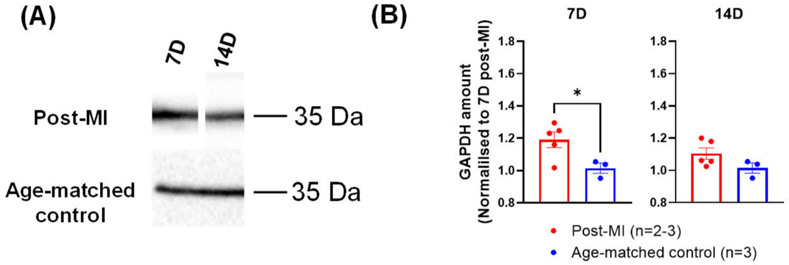

Figure S3: Loading control validation (A) Blot of GAPDH of 7D- and 14D post-MI and age-matched control. (B) Bar graph of GAPDH quantification of post-MI and control groups at respective time points. Unpaired one-tailed t test was performed within each time points. The sample populations are normally distributed, validated by Shapiro-Wilk test. No significant differences were detected in the standard deviations, validated by F-test. 'n' refers to the number mice used for timepoint. *p<0.05

Papillary preparation and experimental set-up for force measurement

Prior to excising the heart, the mouse was anaesthetised under 5% isoflurane/95% O_2 for approximately five minutes. Cervical dislocation was performed when the mouse is deeply anaesthetised which is validated by assessing toe reflexes by pinching the toes. The limbs were fixed with surgical tapes and a pair of sharp/blunt, curved operating scissors was used to create an incision just beneath the ribcage exposing the abdominal cavity. Specifically, the sharp side was used to create the incision. The ribcage was opened by cutting parallel to the sagittal plane at 5mm from the left armpit. This was done using the blunt side to avoid any accidental damage to the myocardium. When the heart was exposed, an 8cm surgical scissors with sharp ends were used to cut the blood vessels connected to the heart. The beating hearts explanted from the mouse were immediately immersed with ice-cold Krebs-Henseleit solution (116 mM NaCl, 4.7 mM KCl, 0.94 mM $MgSO_4$, 1 mM $CaCl_2$, 1.2 mM KH_2PO_4, 25 mM $NaHCO_3$, 11.5 mM glucose) and gently massaged until spurting of blood upon squeeze is ceased (about 10 squeezes) to facilitate the removal of blood from the chambers and vessels. The KH buffer was pre-oxygenated with 95% O_2 and 5% CO_2 for 1 hour and reconstituted with heparin (12 units/ml) and 2,3-butanedione monoxime (BDM, 30 mM/L), prior to explantation.

Finally, the heart was placed on a dissecting dish (coated with silicon polymer at its base) filled with 25mL of ice-cold KH buffer (for 5cm dissecting dish) without heparin and BDM. The buffer temperature was regulated around 4°C on a cooled stage of a stereo dissection microscope. The heart was positioned such that the apex is facing away from the operator and the right ventricle is on the right of the operator. The heart is then pinned down with 0.15mm stainless steel minutien pins at the apex to fix the heart in a stable position, allowing the operator to have a good control of it.

The atria were first removed, exposing the openings of the left and right ventricle chambers. The right ventricle chamber was cut opened by inserting the blade of the scissors into the right ventricle chamber opening (on the right). Right ventricular muscle (RV) are characterised as thin wall and were excised as much as possible. The removal of RV leaves a rough-smooth boundary on the surface. The rough–smooth boundary serves as a useful indicator to cut open the left ventricular chamber without damaging the ventricular papillary muscles. To cut open the left ventricular chamber, the blade of the scissors is placed in the remaining opening and cut about 1mm to the left of the rough-smooth boundary. When the chamber is open and spread, the left ventricle's anterior and posterior papillary muscles connected to the bicuspid valves in the left ventricle can be seen clearly. Part of the left ventricular tissues (spongy texture) was excised, leaving behind the LV tissues connected to the papillary muscles. The excised tissues were transferred to a pre-chilled cryotube and placed in liquid nitrogen to snap-freeze the muscle to retain the phosphorylation level at that point of time. These snap-frozen muscles were stored in -80°C to assay protein phosphorylation levels later.

Anterior and posterior left ventricular papillary muscle was excised and trimmed into a cylindrical shape (200-250 μm in diameter and 1-2 mm in length). T-clips were crimped onto the ends of the papillary segment and the segment was permeabilized by immersion in relax solution with 2% Triton X-100 overnight at 4°C. The setup of the experiment rig was described before [14]. The permeabilized muscle segment was attached to the experimental rig by hooks between a customized force transducer (AE801, Kronex) and a servomotor in a 6-trough stage. Glue consisting of a drop of shellac solution in ethanol was applied to the ends of the muscle segment to minimize the movement during force measurement and the compliance of the damaged ends of the preparations.

Table S4: Force potentiating buffer constituents

	Relax	Pre-Activating	Active
**TES**	100	100	100
**$MgCl_2$**	7.8	6.8	6.5
**Na_2ATP**	5.7	5.7	5.7
**EGTA**	25	0.1	0
**GLH**	5	20	20
**HDTA**	0	24.9	0
**CaEGTA**	0	0	25
**Na_2Creatine Phosphate**	21.2	21.5	21.5

Calcium sensitivity test

The calcium sensitivity test was previously described [108]. Briefly, the muscle segment was transferred into active buffer with different free Ca2+ concentration ranging between 0.16 µM and 32 µM. The segment was immersed in each concentration for 6 min to ensure the isometric force is developed. All experiments were performed at 20°C.

To characterize the Ca2+ sensitivity, the Hill equation is used as below according to the previous report [109].

$$\frac{P}{P_0} = \frac{1}{1 + 10^{h*(pCa_{50} - pCa)}}$$

where P is the tension, P0 is the maximum force, h is the Hill slope (Hill coefficient). pCa is calculated as pCa= -Log10[Ca2+], and pCa50 is the pCa when the force is equal to P0/2. EC50 is the [Ca2+] when force is equal to P0/2. All calculations were done in Prism 7.0 software.

Table S5: Resolving and stacking gel constituents to resolve phosphorylated sarcomeric proteins

Gel constitution	Volume
Resolving gel	
Deionised water	1.08 mL
30% Acrylamide/Bis (29:1) solution	2.475 mL
1.5M Tris/0.4% SDS, pH8.8	1.25 mL
10% SDS	50 µL
10mM $MnCl_2$	25 µL
5mM Phos-Tag in 3% (v/v) Methanol	25 µL
TEMED	7.15 µL
10% APS	35.75 µL
Stacking gel	
Deionised water	1.5 mL
30% Acrylamide/Bis (29:1) solution	0.35 mL
0.5M Tris/0.4% SDS, pH6.8	0.625 mL
TEMED	10 µL
10% APS	12.5 µL

Table S6: Antibodies used for sarcomeric protein and enzymes detection

Targeted Proteins	Product Code/Company	Dilution
**RLC**	Abcam ab92721 (1° Ab)	1:6250
	Abcam ab6721 (2° Ab)	1: 1429
**MyBP-C**	Abcam ab133499 (1° Ab)	1:500
	Abcam ab6721 (2° Ab)	1: 1429
**TnI**	ProteinTech 21652-1-AP (1° Ab)	1:1000
	ProteinTech SA00001-7L (2° Ab)	1:4000
**MLCK**	Abcam ab177358 (1° Ab)	1:2500
	Abcam ab6721 (2° Ab)	1:1429
**ZIPK**	Abcam ab210528 (1° Ab)	1:1250
	Abcam ab6721 (2° Ab)	1: 1429
**MYPT2**	ProteinTech 13366-1-AP (1° Ab)	1:10000
	ProteinTech SA00001-7L (2° Ab)	1:5000

Table S7: Resolving and stacking gel constituents to resolve enzymes

Gel constitution	Volume
Resolving gel	
Deionised water	1.08 mL
30% Acrylamide/Bis (29:1) solution	2.475 mL/1.65mL (15% / 10% gel)
1.5M Tris/0.4% SDS, pH8.8	1.25 mL
10% SDS	50 µL
TEMED	7.15 µL
10% APS	35.75 µL
Stacking gel	
Deionised water	1.5 mL
30% Acrylamide/Bis (29:1) solution	0.35 mL
0.5M Tris/0.4% SDS, pH6.8	0.625 mL
TEMED	10 µL
10% APS	12.5 µL

Expression and purification of Recombinant RLC (rRLC)

The human rRLC was donated from Malcolm Irving's lab at King's College London. It was expressed at 18°C overnight and was induced with 0.3mM IPTG. After expression, cells harvested by centrifugation (20 min, 5000g) were lysed by BugBuster® MasterMix (Novagen) containing EDTA-free protease inhibitor (Sigma). The mixture was then placed on a shaker at room temperature (RT) for 15 minutes. The mixture was centrifuged at 16000g for 20min at 4°C, followed by removal of the supernatant, and this procedure was repeated twice to separate inclusion bodies (IB) from soluble components.

The IB pellet was resuspended in IMAC binding buffer (25mM HEPES, 500 mM NaCl, 1 mM MgCl2, 1 mM DTT, 20 mM imidazole) containing 6 M urea and incubated for 3h at RT on a rocker at 70 rpm. The tubes containing IB were centrifuged at 16000g for 30min at 4°C to remove remaining insoluble components. The supernatant, containing the proteins of interest, was applied to a 5 ml HisTrap FF column (GE Healthcare) with a flow rate of 1–2 ml/min. After washing the column, the protein was eluted with a step gradient of imidazole (50–500 mM, 50mM increments) in IMAC binding buffer containing 6 M urea. After a quick check of the elution profile on SDS-PAGE, fractions containing purified protein were collected, and urea was removed by dialyzing three times against dialysis buffer overnight at 4°C (25 mM HEPES, 250 mM NaCl, 1 mM MgCl2, 1 mM DTT). To remove the N-terminal histidine tag, tobacco etch virus protease was added in a 1:100 stoichiometry and the protein was digested overnight at 4 °C. The tobacco etch virus proteases and remaining impurities were removed by passing through 1 ml HisTrapFF (GE Healthcare) columns. The column flow-through containing the purified rRLC was collected, and the protein was concentrated to 1.5 mg/mL (UFC901024, AMICON spin concentrators). Protein was stored at -80 °C until further use.

RLC exchange efficiency

A **B**

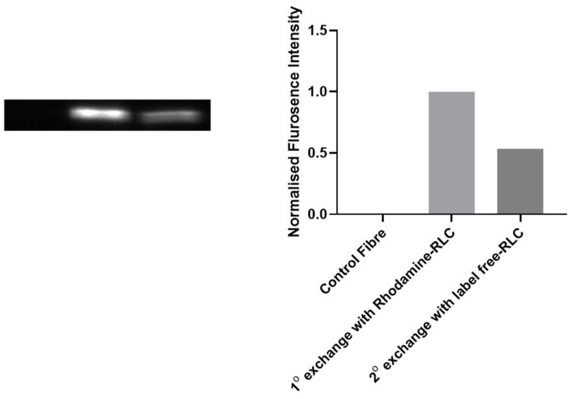

Exchange efficiency

$$= \frac{1°Rho\cdot RLC\ signal - (1°Rho\cdot RLC\&2°unlbl\cdot RLC)\ signal}{1°Rho\cdot RLC\ signal}$$

= 47%

Figure S4: RLC exchange efficiency in cardiac papillary muscle segments (A) Gel image of control rRLCs and Rhodamine labelled-rRLCs. (B) Graph shows normalised fluorescence intensity. Control fibres emit no signal, while the signal drops by almost 50% from primary (1°) exchange with Rhodamine labelled-RLC to 2°exchange with unlabelled RLC (recombinant RLC). Fluorescence signals collectively measured from 5 fibres for each group.
